# Human Gene Expression in Uncomplicated *Plasmodium falciparum* Malaria

**DOI:** 10.1155/2015/162639

**Published:** 2015-09-30

**Authors:** James M. Colborn, Joni H. Ylöstalo, Ousmane A. Koita, Ousmane H. Cissé, Donald J. Krogstad

**Affiliations:** ^1^Center for Infectious Diseases, Tulane University Health Sciences Center, New Orleans, LA 70112, USA; ^2^Department of Tropical Medicine, Tulane University Health Sciences Center, New Orleans, LA 70112, USA; ^3^Centers for Disease Control and Prevention, 1600 Clifton Road, Atlanta, GA 30333, USA; ^4^Center for Gene Therapy, Tulane University Health Sciences Center, New Orleans, LA 70112, USA; ^5^Department of Biology, University of Mary Hardin-Baylor, 900 College Street, Box 8432, Belton, TX 76513, USA; ^6^The Faculties of Science, Technology, Medicine, Pharmacy, and Odontostomatology, University of Bamako, Bamako, Mali; ^7^Institute of Microbiology, University of Lausanne, 1011 Lausanne, Switzerland; ^8^Department of Medicine, Tulane University Health Sciences Center, New Orleans, LA 70112, USA

## Abstract

To examine human gene expression during uncomplicated *P. falciparum* malaria, we obtained three samples (acute illness, treatment, and recovery) from 10 subjects and utilized each subject's recovery sample as their baseline. At the time of acute illness (day 1), subjects had upregulation of innate immune response, cytokine, and inflammation-related genes (IL-1*β*, IL-6, TNF, and IFN-*γ*), which was more frequent with parasitemias >100,000 per *μ*L and body temperatures ≥39°C. Apoptosis-related genes (Fas, BAX, and TP53) were upregulated acutely and for several days thereafter (days 1–3). In contrast, the expression of immune-modulatory (transcription factor 7, HLV-DOA, and CD6) and apoptosis inhibitory (c-myc, caspase 8, and Fas Ligand G) genes was downregulated initially and returned to normal with clinical recovery (days 7–10). These results indicate that the innate immune response, cytokine, and apoptosis pathways are upregulated acutely in uncomplicated malaria with concomitant downregulation of immune-modulatory and apoptosis inhibitory genes.

## 1. Introduction

Malaria is caused by intracellular protozoa of the genus* Plasmodium* [1]. Each year 300 million to 3 billion people are infected and more than 600,000 die, making malaria the most important vector-borne disease in the world [2]. Of the five parasites that infect humans (*P. falciparum*,* P. vivax*,* P. malariae*,* P. ovale*, and* P. knowlesi*),* P. falciparum* causes the most severe disease and the most morbidity and mortality [3]. Its manifestations range from asymptomatic to fatal infection [4, 5] and there are major gaps in our understanding of its pathogenesis [6] and the changes in gene expression responsible for the host response that are the focus of this study [7, 8].

Previous studies have shown that humans with malaria release proinflammatory mediators, such as tumor necrosis factor (TNF), interferon gamma (IFN-*γ*), and nitric oxide (NO), which may damage organs such as brain, lung, and kidney [5], and suggest that the levels of these mediators are related to the severity of disease [9–13]. Potential parasite virulence factors include the glycolipids that anchor parasite proteins to the red cell surface [14, 15],* var* genes [16, 17], and hybrid sequences in parasite proteins on the surface of parasitized red blood cells (Block 2 region of merozoite surface protein 1 [OA Koita: personal communication]).

However, until now, it has been difficult to study host gene expression in malaria because methods available could examine only a few genes at a time (Northern blots, reverse transcriptase PCR). In contrast, cDNA [18] and oligonucleotide microarrays [19, 20] permit examination of the entire human transcriptome. To examine host gene expression in malaria, it was necessary to examine and resolve (1) effects of ambient temperatures > 45°C on RNA stability, (2) transport of RNA preparations from malarious areas to the U.S., (3) potential confounding by host genomic differences and host variation in gene expression, (4) the effects of antimalarial treatment on host gene expression, and (5) potential false-positive and false-negative microarray results.

## 2. Methods

### 2.1. Blood Collection and RNA Isolation

Venous blood samples (3 mL) were drawn from children 3–16 years of age with uncomplicated malaria and transferred to tubes containing Ficoll-Hypaque (CPT Tube 362760, Becton Dickinson). After centrifugation at 1,500 ×g for 20 min at room temperature, peripheral blood mononuclear cells (PBMCs) formed a 1-2 mm layer 5 mm above the gel separator which was aspirated manually (Samco Pipet 335, San Fernando, CA), mixed with 1.3 mL RNA*later* (Ambion, Austin, TX), and stored at 0°C (ice-water bath). Within 2 hours, RNA was isolated from this suspension using the RiboPure-Blood kit (Ambion).

### 2.2. RNA Quality

**Figure 1:**
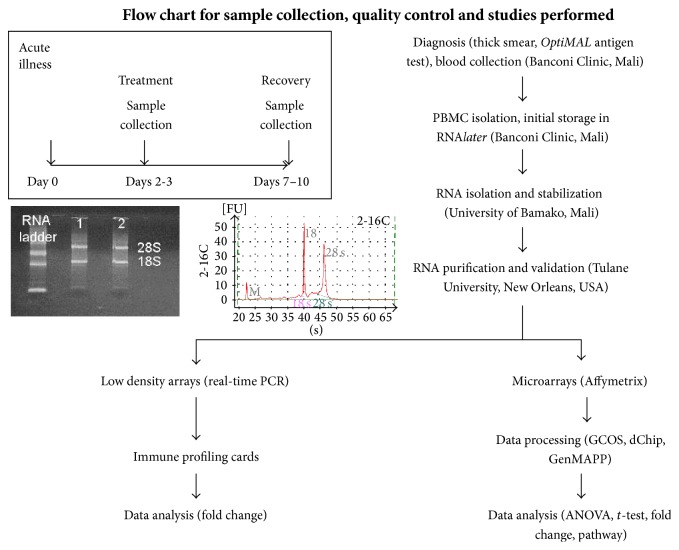
Study design and flow chart for sample collection. The time line (upper left) provides the times when the 3 samples were collected. The insets provide sample results of the procedures used to test RNA quality: agarose gel electrophoresis performed on-site in Mali (left) and capillary electrophoresis using the Agilent Biolanalyzer 2100 in New Orleans (right). Flow chart for sample collection, quality control, and studies performed.

Was assessed by agarose gel (Figure 1) and OD 260/280 ratios in Mali. Samples with distinct 18S and 28S bands on agarose gel and OD 260/280 ratios ≥ 1.7 were stored in sodium acetate : ethanol (0.1 vol 3 M sodium acetate, [pH 5.2] : 2.5 vol 98% ethanol) at −20°C until transport to New Orleans where they were stored at −20°C for ≤ 4 weeks. RNA was purified by ethanol precipitation, followed by cleanup with the RNeasy Kit (Qiagen). RNA was then reexamined using the RNA 6000 Nano Marker Green kit (25–6,000 nt standards, Ambion) and the 2100 Bioanalyzer (Agilent Technologies, Waldbronn, GERMANY) to identify 18S and 28S ribosomal peaks on the electropherogram and estimate the RNA Integrity Number (RIN) [21]. Samples with well-defined 18S and 28S peaks on the electropherogram, ≥1.0 *μ*g RNA, and RINs ≥ 6.5 were converted to biotinylated cRNA and hybridized (Figure 1).

### 2.3. Conversion of mRNA to Double-Stranded Biotinylated cRNA

Double-stranded cDNA was synthesized using the Superscript system (Invitrogen), purified by phenol : chloroform extraction, and concentrated by ethanol precipitation.* In vitro* transcription was used to produce biotin-labeled cRNA (GeneChip* In Vitro* Transcription Labeling Kit, Affymetrix).

### 2.4. Microarray Hybridization

Biotinylated cRNA was cleaned (RNeasy), fragmented, and hybridized on HG-U133 Plus 2.0 chips (Affymetrix). Each chip had > 54,000 probe sets for > 38,000 human genes [22]. After washing, chips were stained with streptavidin-phycoerythrin, amplified with biotinylated anti-streptavidin (Vector Laboratories), and scanned for fluorescence (GeneChip Scanner 3000) using the GeneChip Operating software, version 1.0 (GCOS).

### 2.5. Microarray Data Processing

Fluorescence intensities for perfect match (PM) and mismatch (MM) nucleotides were used to determine whether mRNA for specific genes was present (P), marginally present (M) or absent (A). Scanned images were transferred to dChip [23–25] and corrected for image discontinuities. To compare results across chips, one array was chosen as baseline (147C, median intensity 167) to which others were normalized by calculating expression values based on PMs and MMs. Negative results were assigned a value of one.

### 2.6. Filtering

Was performed to identify genes with changes in expression between (1) acute illness (*A*) and recovery (*C*), (2) acute illness (*A*) and treatment (*B*), or (3) treatment (*B*) and recovery (*C*) based on ≥ 90% confidence in a ≥ 1.5-fold change in fluorescence (*p* < 0.01) and a P-call ≥ 70% for samples with up- or downregulation. Samples were permuted 100 times to estimate the false discovery rate which was 0.0% (median), 90% CI: 16.4–25.2%. The three sets of genes were then combined by removing duplicate genes and genes with redundant probe sets.

### 2.7. Hierarchical Clustering in dChip

Expression values were standardized by adjusting samples linearly to a mean of zero with a standard deviation of one and by determining correlation coefficients (*r* values) for normalized expression (intergenic distances defined as 1 − *r*). Genes with the shortest distances between them were merged into supergenes, connected in dendrograms using lengths proportional to genetic distance (centroid-linkage), and repeated *n* − 1 times to cluster all genes. A similar algorithm was used to cluster the samples [26, 27].

### 2.8. Gene Ontologies

Five patterns of expression on the heat maps were examined for Gene Ontology (GO) term enrichment [28] across the entire microarray by using the exact hypergeometric distribution (significant *p* values < 0.01).

### 2.9. Filtering Based on Parasitemia and Temperature (Table 1)

Was based on acute illness samples and used analysis of variance (*p* values ≤ 0.01 and P-calls ≥ 20%); hierarchical clustering was used to identify patterns of expression and to test for GO term enrichment.

### 2.10. Pathway Analysis and Filtering

Identified genes with differences in expression between acute illness and recovery based on (1) ≥ 90% confidence in ≥ 2-fold changes in expression (up or down) and (2) P-calls of 100% for the samples in which genes were up- or downregulated.

### 2.11. Samples from Uninfected Controls

Included 4 subjects who received chloroquine and one who received placebo (5% dextrose in saline). After normalization based on sample 2405A (median intensity 153), cRNA from these subjects was analyzed similarly using dChip.

### 2.12. Microarray Target Validation

Was performed with TaqMan low density arrays using cDNA prepared from total RNA (iScript kit, Bio-Rad), loaded in low density cards, and run on an ABI 7900 HT real-time thermocycler using 50–100 ng cDNA per well. Data were normalized using endogenous 18S cDNA as the control to calculate fold changes between samples with 95% CIs for genes with valid threshold cycle data for 3 or more of 4 replicates (based on acute illness or recovery samples). Genes with changes in expression that overlapped the baseline sample (based on the 95% CI) were excluded from the subsequent analyses.

### 2.13. Subject Identification, Informed Consent, and Study Design

Identification of infected subjects was performed at the Banconi Clinic in Bamako using Giemsa-stained thick blood smears, a positive parasite antigen test, and PCR for the polymorphic Block 2 region of* msp1* [29]. Informed consent was obtained before enrollment from the parents or guardians of children with uncomplicated* P. falciparum* malaria using a protocol approved by the Mali IRB in Bamako and the Tulane IRB in New Orleans. To control for host genomic variation and variation in gene expression, three samples were obtained from each subject (Figure 1): (1) an acute sample at the time of diagnosis on day 1 before beginning treatment (*A*), (2) a treatment sample on day 3 or 4 after completion of the 3-day course of treatment (*B*), and (3) a recovery sample 7–10 days after treatment (*C*, no signs or symptoms of malaria, no asexual parasites on the thick smear). The recovery sample served as the baseline to which the other samples were compared to identify changes from baseline (normal) gene expression. Relevant clinical data, including parasitemia and temperature (Table 1), were obtained at the time of diagnosis and during follow-up visits.

To control for the effects of antimalarials on gene expression, uninfected healthy controls were identified in the village of Missira in the Kolokani District (160 km NW of Bamako) based on a negative Giemsa-stained thick smear, negative parasite antigen test, and the absence of symptoms or signs of malaria. After obtaining consent, these subjects were enrolled in the study and randomized to receive either chloroquine or placebo (5% dextrose in saline).

## 3. Results

### 3.1. Examination of Gene Expression Profiles

Identified three sets of genes with ≥1.5-fold changes between (1) acute illness and treatment (*n* = 127), (2) acute illness and recovery (*n* = 67), or (3) treatment and recovery (*n* = 57). These three sets of genes (Σ = 251) contained 219 unique genes, which were reduced to 182 by removing redundant probes. Conversely, when these criteria were used with samples from uninfected controls, only one gene was identified, indicating that antimalarial treatment produced no identifiable changes in gene expression. Gene expression patterns in the treatment and recovery groups (B and C) clustered closest to each other, with the acute illness group (A) being most distant. However, those patterns were not observed with the uninfected controls. Hierarchical clustering indicated that the uninfected controls were most similar to the recovery samples (C) and thus the recovery samples provided an accurate profile of gene expression in healthy subjects.

**Figure 2:**
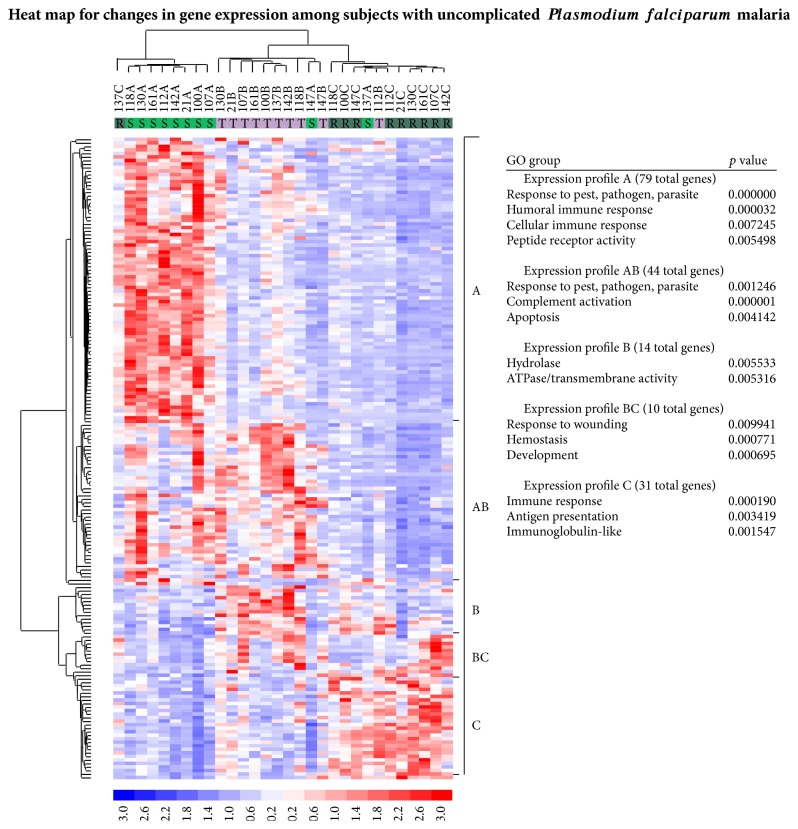
Heat map for changes in gene expression among subjects with uncomplicated malaria. Changes in gene expression with uncomplicated* P. falciparum* malaria revealed five major expression patterns: [A] genes upregulated at the time of acute illness, [AB] genes upregulated at the times of acute illness and treatment, [B] genes upregulated at the time of treatment, [BC] genes upregulated at the times of treatment and recovery, and [C] genes upregulated at the time of recovery. Based on Gene Ontology (GO) categories, profile A and AB genes were related to the immune response, AB genes to apoptosis, and C genes to immune-modulatory functions. In contrast, similar changes in gene expression were not observed in healthy control subjects without* P. falciparum* malaria (data not shown). Heat map for changes in gene expression among subjects with uncomplicated* Plasmodium falciparum* malaria.

Hierarchical clustering of these 182 genes identified five expression profiles, four of which were linked to Gene Ontology (GO) categories such as host immune response or apoptosis (Figure 2). These four profiles included (1) genes upregulated during acute illness (A), (2) genes upregulated during both acute illness and treatment (AB), (3) genes upregulated during both treatment and recovery (BC) and (4) genes upregulated only at the time of recovery (C). The fifth profile (B), which contained genes upregulated only during treatment, was not included in the subsequent analyses because those genes were scattered across Gene Ontology categories.

*Expression profile A (79 upregulated genes, 65 in 41 GO categories)* included genes involved in the innate immune response, effector cell activation, and chemotaxis: leukocyte immunoglobulin-like receptor (LIR) family, neutrophil/monocyte/macrophage chemotactic chemokine receptor-like 2 (CCRL2), toll-like receptor 5 (TLR-5), aquaporin 9, complement component receptor C3AR1, acute-phase cytokine IL-6, and the IL-1*β* receptor antagonist.

*Expression profile AB (44 upregulated genes, 41 in 25 GO categories)* included genes involved in the immune response, apoptosis, and cell death. Immune response genes included complement components C1qA, C1qB, C1qG, and C2, chemokine ligand CXCL16, and Fc III *γ*-receptors CD16a and CD16b. Upregulated apoptosis- and cell death-related genes included BCL2-related X-protein (BAX), BH3 interacting domain death agonist (BID), baculoviral IAP repeat-containing 5 (survivin), and caspase 5.

*Expression profile BC (7 upregulated genes, 7 in 13 GO categories)* included genesinvolved in cellular development and the response to injury such as myosin regulatory protein MYL9, bone morphogenic protein 6, thrombospondin 1, and the oxytocin receptor.

*Expression profile C (31 upregulated genes, 27 in 7 GO categories)* includedgenes upregulated at recovery (10–14 days after acute illness) such as human leukocyte antigens HLA-DOA and HLA-DOB, transcription factor 7, CD6, CD1C antigen, and the Fc IgE receptor, FCER1A.

### 3.2. Pathways (Gene Networks) Involved in Uncomplicated* P. falciparum* Malaria

**Figure 3:**
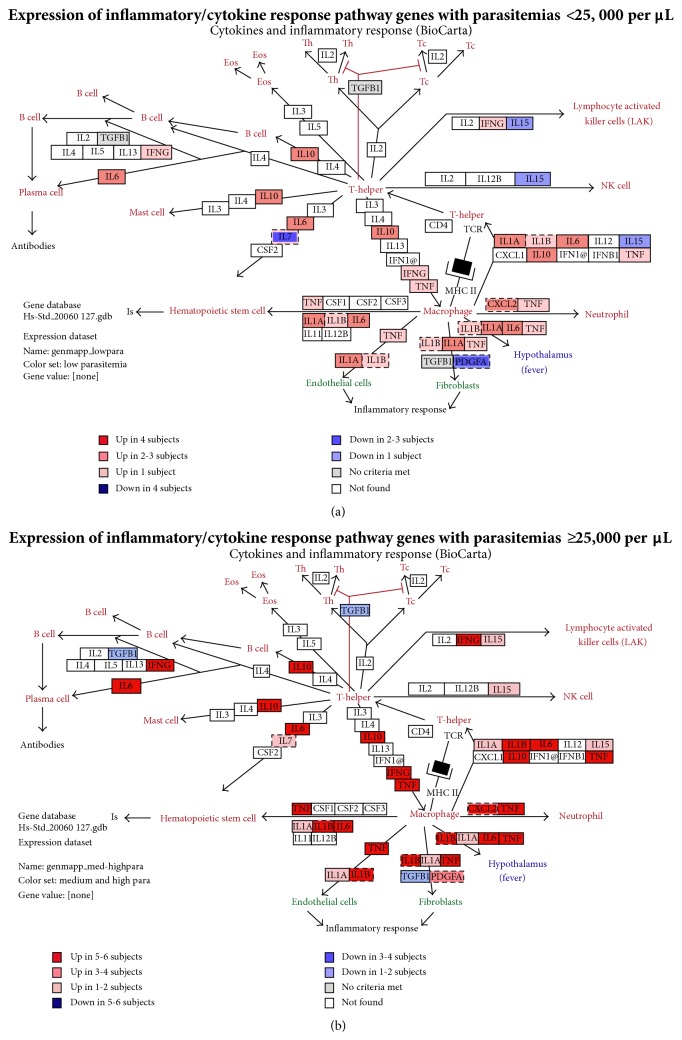
Inflammatory and cytokine response pathways. GenMAPP illustration of genes upregulated with low parasitemias (a) and medium to high parasitemias (b). Several genes were upregulated more frequently with medium or high parasitemias (b) than low parasitemias (a). Among these were IL-1*β*, IL-6, IL-10, TNF, and INF-*γ*. Additionally, a number of genes, including IL-7, IL-15, and PGDFA (platelet-derived growth factor alpha polypeptide), were downregulated with low parasitemias and upregulated with medium to high parasitemias. TGF-*β* was downregulated more with medium to high than low parasitemias.

**Figure 4:**
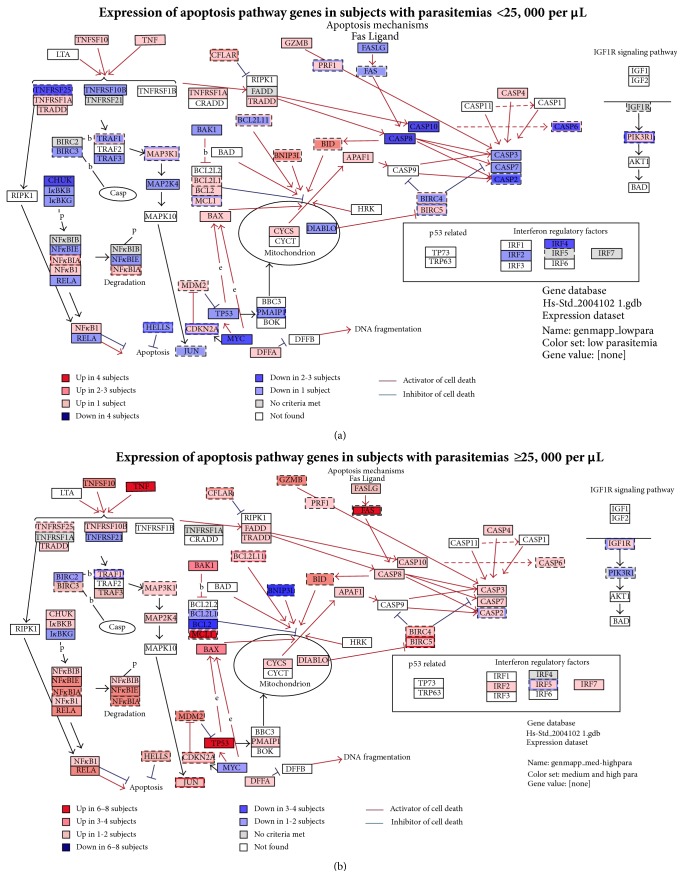
Apoptosis response pathway. Expression of most apoptosis-related genes correlated with the parasitemia. Note that the majority of these genes, including Fas (first apoptosis signal), FasL (Fas Ligand), and TP53 (tumor protein 53), were upregulated in subjects with medium to high parasitemias. In contrast, the BCL2 interacting protein (BNIP) family gene and the BCL2 and BCL2L1 apoptosis inhibitory genes were upregulated in subjects with low parasitemias and downregulated in subjects with medium to high parasitemias.

Based on pathway analyses, a number of cytokine and inflammatory response genes were upregulated, including IL-6, IL-10, IL-1*β*, and TNF (Figure 3). Examination of the apoptosis pathway (Figure 4) demonstrated simultaneous upregulation of genes that facilitate apoptosis (BAX, NF-*κ*1B, TNF SF10, Gzm B, and TNF) and concomitant downregulation of genes that inhibit apoptosis (c-Myc, TNF R SF25, IRF4, KBBKG, caspase 8, and Fas Ligand G).

### 3.3. Pathway Analyses Based on Parasitemia and Temperature

Identified 55 genes based on parasitemia and 21 based on temperature. GenMAPP analyses revealed differences in the inflammatory and apoptosis pathways between subjects with low parasitemias and subjects with medium or high parasitemias. Within the inflammatory, immune response, and host response pathways, the differences were most striking for acute phase mediators such as IFN-*γ*, IL-1*α*, IL-1*β*, IL-6, IL-7, IL-10, IL-15, and TNF (Figures 3(a)-3(b)). Within the apoptosis pathway, the differences were most striking for TP53 (p53), Fas, Fas Ligand, MCL1, caspases 6, 8, and 10, TNF, and TNFSF10B and 25 (Figures 4(a)-4(b)). Similar results were obtained with increasing body temperature (data not shown).

### 3.4. Upregulation of the Inflammatory and Apoptosis Pathways with Higher Parasitemias

Was identified with ANOVA using Kruskal-Wallis. Similar upregulation was observed in subjects with elevated temperatures (< 39°C versus ≥39°C, data not shown).

### 3.5. Microarray Validation Using Low Density Arrays

Quantitative real-time PCR using TaqMan low density arrays demonstrated excellent correlations between the changes observed in the microarrays and low density arrays. The magnitudes of changes (fold increase or decrease) in gene expression in the inflammatory response and apoptosis pathways varied similarly with parasitemia in both the microarrays (Figures 3-4) and the low density arrays (data not shown).

## 4. Discussion 

### 4.1. Study Design, Obstacles, and Potential Confounders

The effects of ambient temperatures > 45°C in Mali on RNA quality were addressed by rapid sample processing, by storage of PBMC preparations at 0°C in RNA*later *until the time of RNA extraction, and by the evaluation of RNA quality on-site in Bamako and again in New Orleans. Confounding by individual variation in gene expression was addressed by using the recovery samples from each subject as their control. This study design also permitted the study of changes in expression over time in pathways that were initially up- or downregulated. Confounding by antimalarial treatment was addressed by demonstrating that antimalarial treatment alone had no effect on gene expression.

### 4.2. Comparison with Previous Reports

These results provide the first information of which we are aware for upregulation of the apoptosis pathway with concomitant downregulation of apoptosis inhibitory genes during acute malaria (Figure 4). In contrast, the upregulation of inflammatory, host response, and proinflammatory cytokine genes observed (TNF, IFN-*γ*) is consistent with previous reports (Figure 3) [5, 7, 9, 10, 15, 23, 24] and there was no upregulation of immune response genes in the uninfected controls (data not shown). Although some immune-related genes were downregulated, those downregulated genes had immune-modulatory functions (Figure 2, profile BC).

### 4.3. Pathway Analyses

Revealed upregulation of cytokine, immune response, inflammatory response, complement activation (Figure 3), apoptosis (Figure 4), and Fas pathways [30–32] in subjects with higher parasitemias (25,000–100,000 per *μ*L and >100,000 per *μ*L, Figures 3-4) and higher temperatures (≥39°C, data not shown).

### 4.4. The Role of the Innate Immune System in Uncomplicated* P. falciparum* Malaria [33–35]

Is supported by the upregulation of Toll-like receptors (TLR 5) [36, 37], NK cell receptors (LILRs, KIRs), and chemokines during the acute illness (day 1). Indeed, several studies have suggested that the glycosylphosphatidylinositol (GPI) lipopolysaccharide which anchors* P. falciparum* proteins to parasite and red cell surfaces may stimulate the production of proinflammatory cytokines such as TNF, and Zhu et al. [15] have shown that GPI induction of IL-6, IL-12, and TNF is dependent on TLR activation via the p38- and NF-*κ*B pathways. In these studies, IL-1*β*, IL-6, and TNF were upregulated more frequently in subjects with higher parasitemias (Figures 3-4) and higher temperatures (data not shown). In addition, studies in Uganda suggest that individuals heterozygous for the TLR 2 Δ22 polymorphism may be protected from cerebral malaria [38].

### 4.5. Apoptosis in Malaria

The data reported here are likewise consistent with previous studies suggesting that apoptosis plays an important role in the pathogenesis of uncomplicated* falciparum* malaria [32, 39–41]. Because they indicate that genes involved in apoptosis, such as Fas, BAX, and TP53, are upregulated during uncomplicated* P. falciparum* malaria* in vivo* (Figure 4), they suggest that apoptosis may be initiated through several different pathways in uncomplicated malaria, including TNF binding to TNFRSF, Fas (which can be induced by TNF), TNF binding to FasL, and possibly HMOX in response to oxidative stress. In these studies, upregulation of apoptosis-related genes correlated with higher parasitemias (>100,000 per *μ*L, Figure 4) and higher body temperatures (≥39°C, data not shown). These results are also consistent with the findings of Wassmer et al. [42] who suggested that TGF-*β*1 from activated platelets may stimulate endothelial cell apoptosis and death in cerebral malaria. Despite the evidence from these and other studies for apoptosis in* P. falciparum* infection, the actual role(s) of apoptosis in malaria remains unclear although a number of studies have reported that this pathway is upregulated in severe malaria [6, 43, 44].

## 5. Conclusions 

The data reported here indicate that the immune response, proinflammatory, and apoptosis pathways are upregulated at the time of acute illness in uncomplicated* P. falciparum* malaria. The apoptosis pathway remains upregulated for several days (days 1–3) before returning to normal. In contrast, immune-modulatory and apoptosis inhibitory genes are downregulated initially (days 1–3) and return to normal by the time of clinical recovery 7–10 days later.

## Figures and Tables

**Table 1:** Baseline information at the time of enrollment: subjects with uncomplicated *P.  falciparum* malaria and uninfected controls.

Subjects			
Age (years)	Sex (M, F)	Temperature (°C)	Parasitemia (per *μ*L)
6	F	37.9	1,400
13	M	38.0	35,575
14	F	38.0	6,100
9	F	38.6	10,625
7	M	39.4	61,125
16	M	39.6	14,675
14	M	39.6	53,350
3	M	39.7	113,000
9	F	40.0	166,500
11	M	40.0	29,525

Uninfected controls			
6	M	36.2	0 (Neg)
4	M	37.2	0 (Neg)
7	F	36.7	0 (Neg
7	F	36.8	0 (Neg)
2	M	36.0	0 (Neg)

## Abbreviations

Abs: Absent
BAX: BCL2 associated X-protein
CI: Confidence interval
GCOS: Gene chip operating system
GO: Gene Ontology
GPI: Glycosylphosphatidylinositol
HG-U133 Plus 2.0: Gene chip for the study of human gene expression
ID: Identification (as in Gene ID Number)
Marg: Marginal
MM: Mismatch
*msp1*: Merozoite surface protein 1
PM: Perfect match
Pres: Present
RIN: RNA integrity number
TNF: Tumor necrosis factor
TP53: Tumor protein 53 (*p53*).
